# Two novel mollusk short-form ApeC-containing proteins act as pattern recognition proteins for peptidoglycan

**DOI:** 10.3389/fimmu.2022.971883

**Published:** 2022-10-07

**Authors:** Jin Li, Shumin Liu, Yang Zhang, Qiuyun Huang, Hao Zhang, Jihua OuYang, Fan Mao, Huiping Fan, Wenjie Yi, Meiling Dong, Anlong Xu, Shengfeng Huang

**Affiliations:** ^1^ Key Laboratory of Biocontrol, Southern Marine Science and Engineering Guangdong Laboratory (Zhuhai), Guangdong Key Laboratory of Pharmaceutical Functional Genes, School of Life Sciences, Sun Yat-sen University, Guangzhou, China; ^2^ Laboratory for Marine Biology and Biotechnology, Qingdao National Laboratory for Marine Science and Technology, Qingdao, China; ^3^ Chinese Academy of Sciences Key Laboratory of Tropical Marine Bio-Resources and Ecology and Guangdong Provincial Key Laboratory of Applied Marine Biology, South China Sea Institute of Oceanology, Chinese Academy of Sciences, Guangzhou, China; ^4^ School of Life Sciences, Beijing University of Chinese Medicine, Beijing, China

**Keywords:** ApeC, ACP, microbial binding, peptidoglycan, mollusca, *Crossostrea gigas*, *Biomphalaria glabrata*

## Abstract

The Apextrin C-terminal (ApeC) domain is a new protein domain largely specific to aquatic invertebrates. In amphioxus, a short-form ApeC-containing protein (ACP) family is capable of binding peptidoglycan (PGN) and agglutinating bacteria *via* its ApeC domain. However, the functions of ApeC in other phyla remain unknown. Here we examined 130 ACPs from gastropods and bivalves, the first and second biggest mollusk classes. They were classified into nine groups based on their phylogenetics and architectures, including three groups of short-form ACPs, one group of apextrins and two groups of ACPs of complex architectures. No groups have orthologs in other phyla and only four groups have members in both gastropods and bivalves, suggesting that mollusk ACPs are highly diversified. We selected one bivalve ACP (CgACP1; from the oyster *Crossostrea gigas*) and one gastropod ACP (BgACP1; from the snail *Biomphalaria glabrata*) for functional experiments. Both are highly-expressed, secreted short-form ACPs and hence comparable to the amphioxus ACPs previously reported. We found that recombinant CgACP1 and BgACP1 bound with yeasts and several bacteria with different affinities. They also agglutinated these microbes, but showed no inhibiting or killing effects. Further analyses show that both ACPs had high affinities to the Lys-type PGN from S. *aureus* but weak or no affinities to the DAP-type PGN from Bacillus *subtilis*. Both recombinant ACPs displayed weak or no affinities to other microbial cell wall components, including lipopolysaccharide (LPS), lipoteichoic acid (LTA), zymosan A, chitin, chitosan and cellulose, as well as to several PGN moieties, including muramyl dipeptide (MDP), N-acetylglucosamine (GlcNAc) and N-acetylmuramic acid (MurNAc). Besides, CgACP1 had the highest expression in the gill and could be greatly up-regulated quickly after bacterial challenge. This is reminiscent of the amphioxus ACP1/2 which serve as essential mucus lectins in the gill. Taken together, the current findings from mollusk and amphioxus ACPs suggest several basic common traits for the ApeC domains, including the high affinity to Lys-type PGN, the bacterial binding and agglutinating capacity, and the role as mucus proteins to protect the mucosal surface.

## Introduction

The Apextrin C-terminal (ApeC) domain is a novel protein domain first discovered in the cephalochordate amphioxus (1–3), a basal living chordate lineage (4). ApeC was later found to be widely distributed in at least fifteen invertebrate phyla, most of which are marine and freshwater invertebrates, including ascidians, cephalochordates, echinoderms, hemichordates, mollusks, cnidarians, etc (5). However, ApeC was not found in vertebrates and nor in the major arthropod lineages including insects and crustaceans, which may make ApeC largely an aquatic invertebrate-specific protein domain (5). ApeC is a large domain with about 200 amino acids, including eight conserved cysteines and three conserved DEXD motifs, and is mostly present in secreted and membrane-bound proteins (5). ApeC are found to form different domain combinations with other domain types (5), making it one of the versatile and promiscuous domains like those immunoglobulin (IG) and C-type lectin domains.

Thus far, the functional information of ApeC domains came from a unique ApeC-containing proteins (ACPs) family in amphioxus. Four members (ACP1, 2, 3 and 5) of this family were investigated (2, 6). Despite the sequence and structural differences, these ACPs adopt a core architecture: a leading signal peptide (SP) and a C-terminal ApeC domain. They could agglutinate bacteria by using the ApeC domain to bind with the cell wall component peptidoglycan (PGN), but they showed no inhibiting or killing effects on bacteria. ACP1 and ACP2 are mainly expressed in the gill and skin, while ACP3 and ACP5 are concentrated in the gut. Secreted ACP1 is indispensable for the anti-bacterial mucosal immune responses in the gill, while ACP2 and ACP3 can regulate the TRAF6-NF-κB pathways when present in cytosol. Therefore, this amphioxus ACP family have dual roles, as extracellular lectins for PGN and as intracellular immune regulators (2, 6).

In other phyla, the functions of ApeC domain have not been directly investigated, but there are evidence suggesting that many ACPs might have important roles in development, immunity and stress resistance. In the sea chitin *Heliocidaris erythrogramma*, an ACP called apextrin is a secreted protein with a unique MACPF/perforin-ApeC domain structure (7–9). During embryogenesis, this protein was found to be highly expressed and concentrated in the apical extracellular matrix of ectoderm columnar cells, hence was suggested to be involved in apical cell adhesion. Another apextrin, from the clam *Ruditapes philippinarum*, exhibited an inhibitory effect on gram-positive bacteria probably due to its MACPF/perforin domain, but the role of its ApeC domain was not examined (10). In mussel *Mytilus galloprovincialis*, two ACPs called apelB and apelP, were also proposed to have a role in embryogenesis because their expression could be increased thousands of times after fertilization and reached a peak at the blastula and trochophore stages (11). In the oyster *Crossostrea gigas*, an ACP was found to be highly up-regulated under hypoxic conditions, suggesting a role in the anti-stress responses (12). In terms of immune roles, several ACP genes from sea urchins, oysters, mussels, clams, and brachiopods exhibited high expression in the immune cells and tissues and could be up-regulated in response to bacterial challenge (11, 13–18). Moreover, a recent study found that ACPs existed in the pedal mucus secreted by limpets, probably being involved in immunity and glue-like adhesion (19).

With approximately 200 thousand extant species, mollusks comprise one of the largest animal phyla, second only to the arthropods (20) and accounting for roughly 10% of the animal biomass (21). Mollusks are heterogeneous in size, morphology, adapting to different habitats (terrestrial, fresh water and marine) and feeding behaviors, hence reflecting its massive radiation since the Cambrian era (22). Bivalvia and Gastropoda represent the two largest mollusk classes. Bivalves are restricted to aquatic life, accounting for about 14% of mollusks, while gastropods comprise over 80% of mollusks, adapted to both aquatic and terrestrial life (23). Previous studies showed that bivalves and gastropods have a large number of ACPs (5). Some mollusk ACPs appear to play roles in different processes including embryogenesis, anti-stress responses, mucus-based adhesion, and mucosal and humoral immunity (11, 12, 15–19).

As a new protein domain, the functions of ApeC remains largely elusive. Previous analysis of an amphioxus ACP family suggests that its members serve as novel pattern-recognition proteins and its ApeC domains are responsible for the carbohydrate-binding activity (2, 6). It is not known if these functional properties could be applied to other ACP families and other phyla. After all, ApeC sequences are highly diverged and no ACP orthologs could be found between different phyla (5). On the other side, mollusks have great ecological and economical significance, and coincidently possess many ACPs appearing to have relevant functions. Therefore, this study is focused on the mollusk ACPs. First, we analyzed their composition, species distribution, phylogenetics, domain architectures and sequence evolution. Then, we chose two highly-expressed, secreted short-form ACPs, one from bivalves and the other from gastropods, for molecular functional experiments. Finally, we compare the functional traits between these two mollusk ACPs and the previously studied amphioxus ACPs. Our findings could provide new insights into the basic ApeC functions, which may help the future functional studies of the ACP members in mollusks and other invertebrate phyla.

## Materials and methods

### Animals, pathogen challenge and hemocyte preparation

The Pacific oysters *Crossostrea gigas* (two ages with an average shell length of 100 mm) were from Qingdao, Shandong Province, China, and were maintained under previously described conditions (24), 22-25°C in a tank with circulating seawater for two weeks prior to the experiment. Oysters were fed twice daily on *Tetraselmis Suecica* and *Isochrysis Galbana*. All experimental manipulations were performed in accordance with local guidelines for the care and use of laboratory animals.

For monitoring the gene expression changes after immune stimulation, bacterial challenged oysters were prepared as previously described (25, 26). Briefly, *Vibrio parahaemolyticus* was cultured in LB (Luria-Bertani) broth at 37°C to OD600=0.6-0.8 (they grew faster at 37°C and remained healthy), and then centrifuged at 800×g for 10 min at 4°C. After being washed for 3 times in PBS (pH 7.4), bacterial pellet was resuspended in PBS to an adjusted density of OD600 = 1.0. Oysters in the challenged group were injected with a 100 μl suspension into the adductor muscle, while oysters in the control group were injected with an equal volume of PBS. After injection, oysters were returned to separate tanks for subsequent sampling.

Oyster hemolymph was extracted from the posterior adductor muscle of the oysters by using a medical-grade syringe (0.45×15.5 mm) and hemolymph from each individual was counted toward one sample. Hemolymph was collected immediately by centrifugation at 200×g for 10 min, then the supernatant was sterilized through a 0.22 µM filter for subsequent analyses. For each time point after immune stimulation, three individuals were sampled and the sampled materials were pooled together for later use.

### RNA isolation and cDNA synthesis

Total RNA was extracted from frozen tissue with TRIzol reagent (Invitrogen) according to the manufacturer’s directions and precipitated with isopropyl alcohol. After quality checks using a spectrophotometer and agarose gel electrophoresis, the RNA was reverse-transcribed to synthesize the first-strand cDNA using the PrimeScript First-strand cDNA Synthesis Kit (Takara) using the oligo d(T) primer following the manufacturer’s protocol. Then the cDNA was stored at -20°C.

### Cloning and synthesizing of the ACP cDNA

Mollusk ACP sequences were obtained by conducting homology-based searches on the National Center for Biotechnology Information database (NCBI, http://www.ncbi.nlm.nih.gov/genbank/) using the amphioxus ApeC sequences as baits. Pacific oyster CgACP1 was identified in the *Crossostrea gigas* genome and the accession is XP_011420902 (or XP_034306861). Gastropod snail BgACP1 was identified in the *Biomphalaria glabrata* genome and the accession is XP_013078768. To obtain the complete cDNA sequence of CgACP1, a pair of gene-specific primers (forward primer: 5’-TTTAACGGTCTAAAGGTCCTG-3’; reverse primer: 5’-AAGAGATTACACATTTGCCT-3’) was used. As for the cDNA sequence of BgACP1, it was directly synthesized based on a manually curated sequence (namely, the consensus sequence of multiple sequences and RNA-seq reads). Finally, the cDNA fragments were cloned into the pGEX-Teasy vector (Promega) and verified by sequencing at The Beijing Genomics Institute (BGI).

### Bioinformatic analysis

The protein domain and signal peptide were predicted with the Simple Modular Architecture Research Tool (SMART, http://smart.embl-heidelberg.de/) and SignalP (http://cbs.dtu.dk/services/SignalP) software, respectively. The isoelectric point (pI) and m.w. were speculated on ExPASy Web site (http://www.expasy.org/tools/). BLASTP was performed to analyze the sequence identities. Multiple sequence alignments of ApeC domains from different species were analyzed using the default parameters of MEGA-X (27) by using ClustalW algorithm and were manually corrected using GeneDoc software (28). The Neighbor-joining phylogenetic tree (29) was built using MEGA-X (27) with the JTT matrix-based method (30) and handling gaps by pairwise deletion. The optimal tree is shown. The percentage of replicate trees in which the associated taxa clustered together in the bootstrap test (1000 replicates) are shown next to the branches.

### Real-time quantitative RT-PCR

Real-time quantitative RT-PCR (QPCR) was performed on the Roche LightCycler 480 using SYBR^®^ Green Realtime PCR Master Mix (Toyobo) according to the manufacturer’s protocol. The reaction volume was 10 µl, which containing 1 µl template cDNA, a primer concentration of 0.5 µM and 5 µl of 2×SYBR Green Mix. The PCR program is 95°C for 1 min, followed by 40 cycles of 95°C for 15 s, 60°C for 15 s, and 85°C for 20 s. The cycle threshold values were calculated by the Roche LightCycler 480 software. The expression levels of different tissues were calculated using 2^-ΔCt^, and the fold change after challenge were calculated using 2^-ΔΔCt^ method based on the cycle threshold values. Reaction of each sample was performed in triplet using *gapdh* mRNA as the internal control. *CgACP1* primers for QPCR were 5’-GCCGTGCTAAGTCTCT-3’ (forward) and 5’-TCCAGTGTCCTCATTATCC-3’ (reverse), and *gapdh* primers for QPCR were 5’-CTTTCCGCGTACCAGTTCCA-3’ (forward) and 5’-GCTGCTTCGCTTGTCTCCAC-3’ (reverse).

### Transcriptome analysis

Three mollusk species, including *Aplysia californica*, *Biomphalaria glabrata* and *Crossostrea gigas*, have high-quality genome assemblies and abundant transcriptome data deposited in the NCBI server. The accumulated expression levels (total RNA-seq raw read counts) of their short-form ACP genes could be queried through NCBI’s Genes of Genomes in the Reference Sequence Collection (https://www.ncbi.nlm.nih.gov/gene/). For comparison, we also analyzed the short-form ACP genes in six species from other four phyla, including *Echinodermata*, *Hemichordata*, *Cephalochordata* and *Cnidaria*.

To analyze the expression of CgACP1 in different tissues and at different time points in the gill after immune stimulation, we downloaded both the pacific oyster (*Crossostrea gigas*) reference genome (assembly oyster_v9: GCF_000297895) and its corresponding gene annotation files from NCBI’s genome website. The RNA-seq data in different tissues were downloaded from NCBI’s databases (BioProject PRJNA146329) (31). The Transcriptome data of the Pacific oyster under *Vibrio* (an equal mixture of *V. anguillarum, V. tubiashii, V. aestuarianus, V. alginolyticus*) challenge at different time points were also downloaded from NCBI’s databases (BioProject PRJNA194079) (32). The Raw RNA-seq data was cleaned using Trimmomatic v.0.36 (33) with default parameters for quality control. The clean data was mapped to the oyster reference genome using Hisat2 v.2.1.0 (34) under default parameters. The fragments per kilobase of exon model per million mapped fragments (FPKM) of each gene was calculated.

### Preparation of recombinant proteins

Recombinant Mollusk ACP proteins were expressed in two different processes. PCR fragments encoding mature amino acids 21-435 aa of BgACP1 and 21-239 aa of CgACP1 were amplified by recombinant primers from the pGEM-T Easy vector (Promega) carrying full-length cDNA and inserted into plasmid pET32a (+) by recombinant method mentioned in ClonExpress^®^II One Step Cloning Kit (Vazyme). BgACP1 recombinant primers were 5’-gctgatatcggatccTCATTCTTGGTGACCATCAACCC-3’ (forward) and 5’-cgaattcggatccgaAACTCGTTCTTCGTAATAGCACAGC-3’ (reverse); CgACP1 recombinant primers were 5’-gctgatatcggatccGTCGGATGGCCTTCTGGAA-3’ (forward) and 5’cgaattcggatccga-ACTGTAGTAGCAGTAGTAGAGACGATGGT (reverse). Recombinant plasmids were confirmed by DNA sequencing to construct the expression vector. Then, the expression plasmids were introduced into *E. coli* BL21 (DE3) and 0.1mM Isopropyl β-D-1-thiogalactopyranoside (IPTG) was added to induce the protein expression. TRX-BgACP1 was solube and expressed in the supernatant but TRX-CgACP1 was insoluble and expressed as inclusion body. For TRX-BgACP1, the supernatant of cell lysates was applied to a Ni^2^-chelating Sepharose column (GE Healthcare), pooled by elution with 250 mM imidazole. The purified recombinant TRX-BgACP1 was put into dialysis bags and dialyzed in PBS buffer at 4°C for 12 hours three times, and concentrated by filtration through an Ultrafree centrifugal filter device (Millipore).

TRX-CgACP1 fusion protein in inclusion body was denatured in 8 M urea using a Ni^2^-chelating Sepharose column and eluted with 250 mM imidazole. Purified TRX-CgACP1 in 8M urea was renatured by three dialysis steps as previously described (35) with certain modifications, first in 3 M urea in PBS buffer containing 150 mM NaCl, pH 7.4, 2 mM reduced glutathione, 0.02 mM oxidized glutathione, 5% glycerol, and 0.05% Tween-20, then in 1 M urea in the same buffer, and finally in the buffer without urea, glutathione and Tween-20. Each dialysis step was performed for at least 12 hours at 4°C. Soluble fusion protein was concentrated by filtration through an Ultrafree centrifugal filter device (Millipore). The protein concentration was determined using the Pierce™ BCA Protein Assay Kit (Thermo) according to the manufacturer’s protocol.

### Microbial binding assays

The bacteria including *Staphylococcus aureus*, *Enterococcus faecalis*, *Escherichia coli*, *Vibrio anguillarum*, *Vibrio parahemolyticus*, *Acinetobacter calcoaceticus* and *Saccharomyces cerevisiae* were inoculated and cultured as described (6). The cells were washed and resuspended in PBS buffer. Approximately 2×10^6^ microbes were incubated with 1 μg of purified recombinant proteins in PBS by gentle orbital rotation overnight at 4°C. Microbes were pelleted and washed five times with 1 ml of PBST (0.05%Tween-20 in PBS). The washed pellets were then suspended with reducing sample buffer quickly and denatured by heating at 100°C for 10 min. The binding proteins were validated by Western blot with an anti-His mouse monoclonal antibody (Sigma).

### Microbial aggregation assays

Fluorescein isothiocyanate (FITC, sigma) was used to label bacteria. Microbes collected from liquid cultures were suspended in 1 ml PBS and mixed with 50 μl fluorescein isothiocyanate (FITC) (sigma, 10 mg/ml in DMSO). The reaction was gently agitated at room temperature in the dark for 3h and then washed five times with PBS. FITC-labeled *S. aure*us (2 × 10^8^ cells/ml), *E. faecalis* (2 × 10^8^ cells/ml), *E. coli* (8 × 10^8^ cells/ml), *V. anguillarum* (2 × 10^8^ cells/ml) or *S. cerevisiae* (3 × 10^7^ cells/ml) were mixed with 5 μg TRX fusion proteins in PBS and incubated at room temperature in the dark for 2h, respectively. The agglutinating reaction was examined immediately under fluorescence microscopy (Carl Zeiss).

### Antimicrobial activity assays

The growth curves of *S. aureus* and *E. coli* cultured with recombinant ACP proteins were tested as follows. Two single colonies were picked up and transferred into 1 ml of Luria-Bertani (LB) respectively and grown to mid-log phase. A volume of 50 μl of cell suspension was mixed with 200 μg purified recombinant proteins or TRX and added to 1 mL broth. Each example was incubated with 200 rpm at 37°C and the OD600 was measured every hour.

For colony counts assays, our procedures paralleled previously reported (36). *S. aureus* were suspended and experiments were performed in PBS buffer (pH 7.4). Incubate mixtures contained 10^5^ bacterial CFU and 20 μg ACP proteins or TRX (as negative control) in 100 μl PBS buffer. After incubation for 3 hours at 37°C, 900 μl of the same buffer was added, and two additional serial 10-fold dilutions were made with that buffer. 100 μg portions from the three serial dilutions were spread over LB agar plates. After incubation for 12 hours, colony forming units (CFUs) were counted.

### Binding assays of ACPs with the components of microbes

ELISA was used to analyze the binding of ACP proteins with soluble microbial cell wall components as previously described (37). In brief, a total of 20 μg of peptidoglycan (PGN) from *S. aureus* (Sigma), PGN from *B. subtilis* (Sigma), lipopolysaccharide (LPS, Sigma), lipoteichoic acid (LTA, Sigma), Zymosan A (Sigma), Chitin (Sigma), Chitosan (Sigma), Cellulose (Sigma), muramyl dipeptide (MDP), N-acetylglucosamine (GlcNAc, Sigma) and N-acetylmuramic acid (MurNAc, Sigma) in PBS (PGN, Zymosan A, Chitin, Chitosan and Cellulose were ultra-sonically solubilized) were used to coat a 96-well microplate (Corning 96-well Clear Polystyrene High-Bind Strip well Microplate) for 3 h at 37°C. Nonspecific binding to the wells was prevented by the addition of PBST (0.05% Tween 20 in PBS) containing 10% (wt/vol) skimmed milk overnight at 4°C. Several concentrations of ACP proteins were then added to the well and the mixtures were incubated for 2 h at 37°C. Binding proteins were detected with anti-His mAb (sigma) diluted 10000-fold for 1h at room temperature, followed by an hour incubation with a 5000-fold dilution of HRP-labeled anti-mouse IgG. Between each incubation step, unbound protein, mAb, or HRP-labeled anti-mouse IgG was washed off three times. Incubate with 100 ul TMB Substrate Solution (Thermo) at 25°C for 15 minutes and stop reaction. Add 50 μl of 2M sulfuric acid to each well to stop the reaction, and the absorbance was read at 450 nm. The assay was repeated at least three times.

### Statistical analysis

Quantitative data are presented as the mean ± SD and compared statistically by two-tailed Student *t* test. In all cases, differences of p < 0.05 were considered significant. *p < 0.05, **p < 0.01, ***p< 0.001 indicate statistical differences. All experiments were repeated at least three times.

## Results

### Phylogenetic analysis of mollusk ACPs

**Figure 1:**
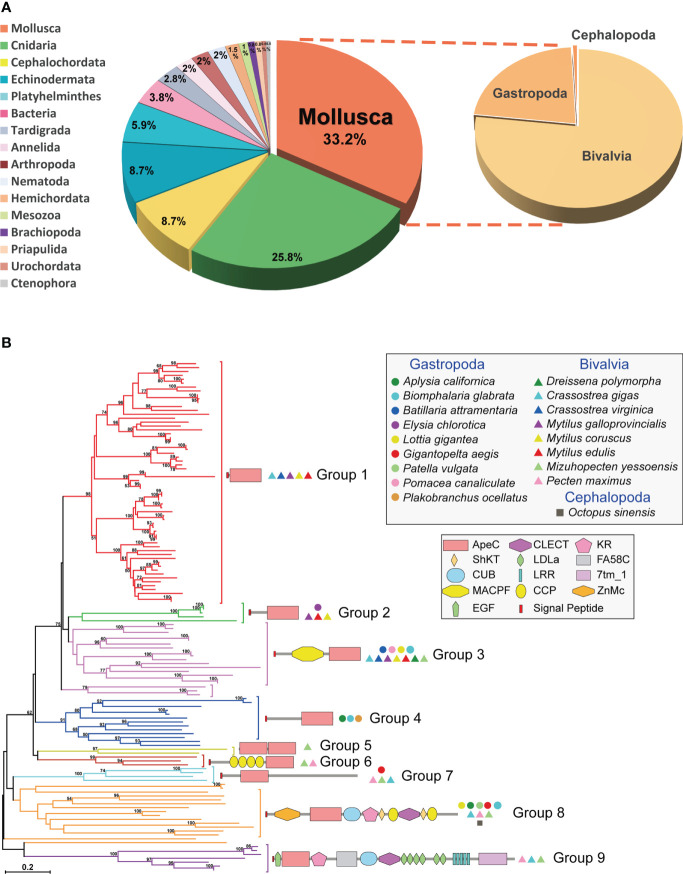
The distribution, phylogenetics and domain architectures of 130 mollusk ACPs. **(A)** The proportion of ACPs in different phyla. **(B)** A phylogenetic tree of all 130 mollusk ACPs based on the ApeC domain. The tree was constructed using the neighbor-joining method. The percentage of replicate trees in which the associated taxa clustered together in the bootstrap test (1000 replicates) are shown next to the branches. The protein architectures, grouping and source species are shown in corresponding positions. A Maximum Likelihood tree based on the same alignment is also provided in **Supplementary Figure 1**. A full list of these mollusk ACPs is provided in **Supplementary Table 1**.

Here we identified 130 non-redundant mollusk ACP sequences from different online databases. One third of these ACP sequences are contributed by mollusks (**Figure 1A** and **Supplementary Table 1**). Each mollusk species has 1-25 ACP sequences, and on average bivalves appear to have more ACPs than gastropods. Particularly, bivalve *Mytilus galloprovincialis* had the largest number of ACPs (up to 25 ACPs), followed by bivalve *Crassostrea gigas* (21 ACPs). As comparison, amphioxus and cnidarians have 2-34 ACPs per species. We conducted a phylogenetic analysis of these sequences using the Neighbor-Joining (NJ) method and the Maximum Likelihood (ML) method (**Figure 1B** and **Supplementary Figure 1**). Based on the phylogenetic trees, mollusk ACPs could be classified into nine groups. None of these groups have orthologs in other phyla, which is consistent with the previous report that ACPs are rapid evolving and highly diversified (5). As comparison, bivalve ACPs have members in eight groups, while gastropod ACPs are only present in five groups. And only four groups (2, 3, 7 and 8) are conserved in both bivalves and gastropods. Moreover, the group 1 is the largest group and contains only bivalve ACPs, hence indicating a bivalve-specific family expansion and diversification. These findings suggest that mollusk ACPs are more diversified and more actively duplicated in bivalves than in gastropods.

### Domain architectures of mollusk ACPs

Mollusk ACPs can be classified into at least nine protein architectures, each corresponding to one phylogenetic group (**Figure 1B**). It suggests that these architectures have their own origins and evolve independently. The group 1, 2 and 4 are secreted short-form ACPs, which features a single C-terminal ApeC domain and no other discernable known domains or motifs. On the other hand, the group 8 and 9 are highly complex proteins, containing more than ten domains or motifs, most of which are often present in extracellular proteins and matrix proteins. The other four groups have medium-sized architectures. Among them, the group 6 features a dual-ApeC module. This architecture is also found in other phyla, including Cnidaria, Tardigrada, Echinodermata and Platyhelminthes (5), though they share no orthologous relationships. Unlike other short repetitive motifs, ApeC is a large domain with ~200 aa, therefore we suspect that this dual-ApeC module may represent a conserved but unknown functional mode. As comparison, versatile immunoglobulin (IG) and C-type lectin domains are often present in tandem repeats. Moreover, the group 3 contains a special group of ACPs called apextrins. This subfamily adopts a highly conserved architecture (signal_peptide + MACPF/perforin+ApeC), and exist in at least four other phyla, including Cnidaria, Tardigrada, Hemichordata and Echinodermata, although no orthologs can be found between phyla. Notably, it has been suggested that in sea urchins and clams, apextrins participated in embryogenesis and immunity, respectively (7–10). Taken together, mollusk ApeC domains are present in various protein architectures.

### Characterization of two short-form ACPs from oysters and snails

**Figure 2:**
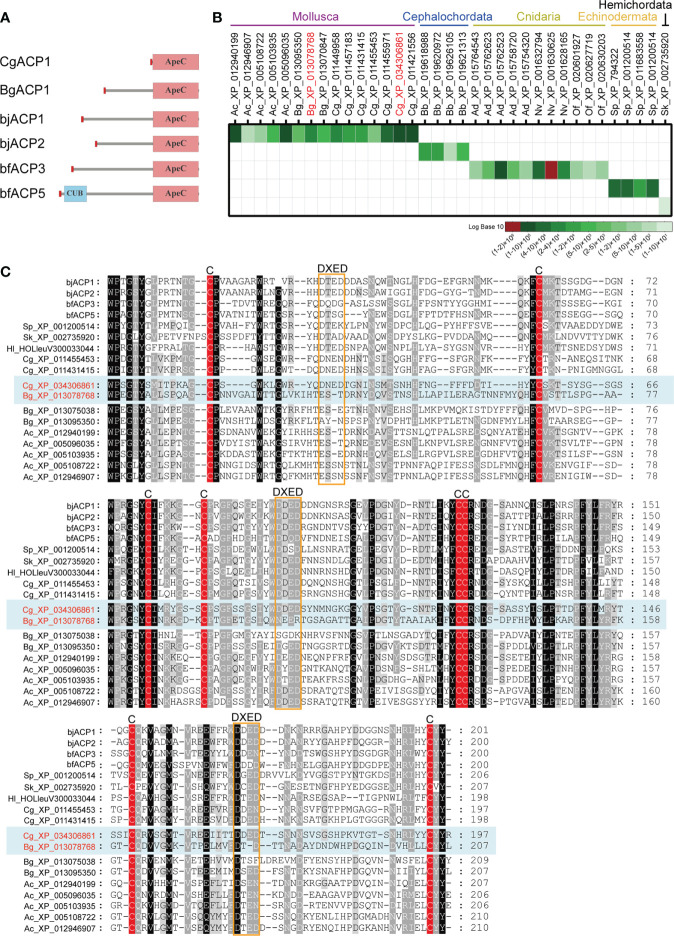
Comparison of the amino acid sequences and the global expression levels of a set of representative ACPs. **(A)** The domain architectures of the oyster CgACP1 and the snail BgACP1 compared with the amphioxus ACP1/2/3/5. **(B)** The accumulated expression level of a set of representative ACPs based on the RNA-seq data from NCBI’s gene browser. The accession for CgACP1 and BgACP1 are highlighted in red. The raw expression data are shown in **Supplementary Table 2**. **(C)** A multiple alignment of the ApeC domains of several short-form ACPs selected from different aquatic invertebrates. The conserved cysteine residues and the DXED motifs are highlight by red color and orange boxes, respectively. The accession of CgACP1 and BgACP1 are also highlighted in red. Ac, *Aplysia_californica*; Bg, *Biomphalaria glabrata*; Cg, *Crossostrea gigas*; bb, *Branchiostoma belcheri*; bj, *Branchiostoma japonicum*; bf, *Branchiostoma floridae*; Hl, *Holothuria leucospilota*; Ad, *Acropora digitifera*; Nv, *Nematostella vectensis*; Of, *Orbicella faveolata*; Sk, *Saccoglossus kowalevskii*; Sp, *Strongylocentrotus purpuratus*.

To understand the basic functions of mollusk ApeC domains, we chose two ACPs for experiments based on these criteria: having a short-form architecture and the high expression level. These criteria were previously used to identify the amphioxus ACP1 and ACP2 (2). The first ACP (CgACP1) is chosen from the pacific oyster *C*. *gigas*, which is a representative bivalve, a famous economic species and a model organism for the mollusk immune and anti-stress systems (38). CgACP1 belongs to the group 1 of ACPs, which contains the shortest form of ACPs ever known and has no homologs from the gastropods (**Figures 1B**, **2A**). For the purpose of comparison, we also selected a gastropod ACP (BgACP1) from the freshwater snail *Biomphalaria glabrata*, which is the intermediate host for the pathogen of human intestinal schistosomiasis (39). BgACP1 belongs to the group 4 ACPs, which has a short-form architecture and contains no homologs from the bivalves (**Figures 1B**, **2A**). According to the accumulated RNA-seq statistics in NCBI’s gene portal, both CgACP1 and BgACP1 have the highest expression among all ACP genes in their own species (**Figure 2B** and **Supplementary Table 2**). As comparison, amphioxus ACP1 also have the highest expression among all amphioxus ACPs (2).

We cloned the full-length cDNA of CgACP1. It encodes a 219 aa protein, which contains only a signal peptide and an ApeC domain, hence is the shortest and simplest ACP ever found (**Figure 2A**; full sequence shown in **Supplementary Figure 2A**). The ApeC domain of CgACP1 has about 47% similarity with the previously reported amphioxus ACP1/2/3. As for BgACP1, we did not have access to the animal of *B. glabrata*, hence its coding DNA was directly synthesized. BgACP1 has 435 aa, containing a signal peptide, a short middle region and a C-terminal ApeC domain (**Figure 2A**; full sequence shown in **Supplementary Figure 2B**). The length and structure of BgACP1 is similar to amphioxus ACP1/2/3, but its shares only 35% identity with amphioxus ACP1/2/3 in the ApeC domain. We created an ApeC-based multiple alignment for a selected set of short-form ACPs from different phyla (**Figure 2C**). It shows that both CgACP1 and BgACP1 have all eight conserved cysteines, which is the most prominent feature of the ApeC domains in all animals. In addition, CgACP1 preserve all three DXED motifs, while BgACP1 has mutations in all three DXED motifs.

### The expression patterns of CgACP1

**Figure 3:**
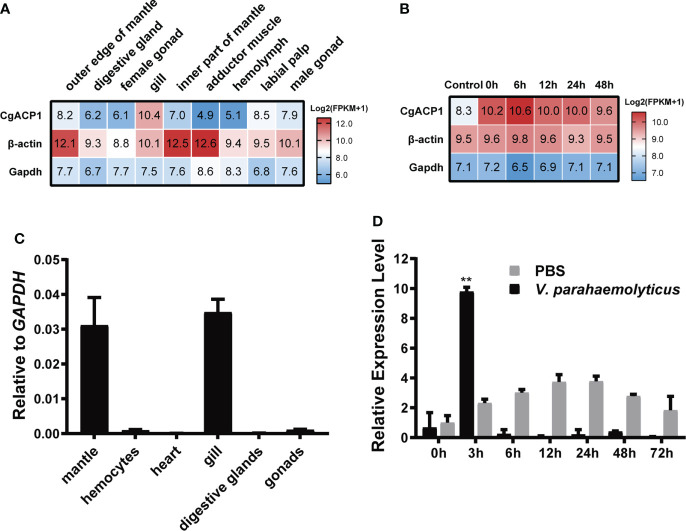
The expression patterns of the CgACP1 gene. **(A)** The *CgACP1* mRNA expression in different tissues based on the RNA-seq data from NCBI BioProject PRJNA146329 (31). The raw expression data are provided in **Supplementary Table 3**. **(B)** The *CgACP1* mRNA expression in the gill before and after *Vibrio* challenge based on the RNA-seq data from NCBI BioProject PRJNA194079 (32). The raw expression data are provided in **Supplementary Table 4**. **(C)** Real-time quantitative RT-PCR analysis of the relative mRNA expression of *CgACP1* in different tissues. **(D)** Real-time quantitative RT-PCR analysis of the relative mRNA expression of *CgACP1* in the hemocytes before and after *Vibrio* challenge. The qPCR-based expression data were shown as a ratio to the *gapdh* mRNA expression and were plotted as the mean ± SD. **p < 0.01 versus mRNA level at 0 h.

CgACP1 has been shown to be the highest expressed ACP in pacific oysters (**Figure 2B** and **Supplementary Table 2**). Here its expression in different adult tissues was further evaluated. Using the public available transcriptome data (32, 33), we showed that CgACP1 was predominantly present in the gill and labial palp, at an expression level even higher than that of beta-actin (**Figure 3A** and **Supplementary Table 3**). Note that the gill and labial palp are adjacent to each other and comprise the filter feeding organ. This expression pattern was confirmed by quantitative RT-PCR assays (**Figure 3B**). Transcriptome data also indicate that the expression of CgACP1 in the gill could be greatly up-regulated shortly after being challenged by a mixture of *Vibrio* bacteria (**Figure 3C**). On the other hand, the hemolymph and the abductor muscle, had the lowest expression of CgACP1, approximately 1/50 of the expression in the gut (**Figure 3A** and **Supplementary Table 3**). Quantitative RT-PCR assays further showed that even after *Vibrio* bacterial stimulation, the expression of CgACP1 in the hemolymph was only transiently up-regulated (**Figure 3D**). This is surprising because the hemolymph contains mainly the immunocompetent hemocytes. Anyway, these findings suggest that CgACP1 should have a more important role in the gill than in the hemocytes.

### Recombinant CgACP1 and BgACP1 bound and aggregated different microbes

**Figure 4:**
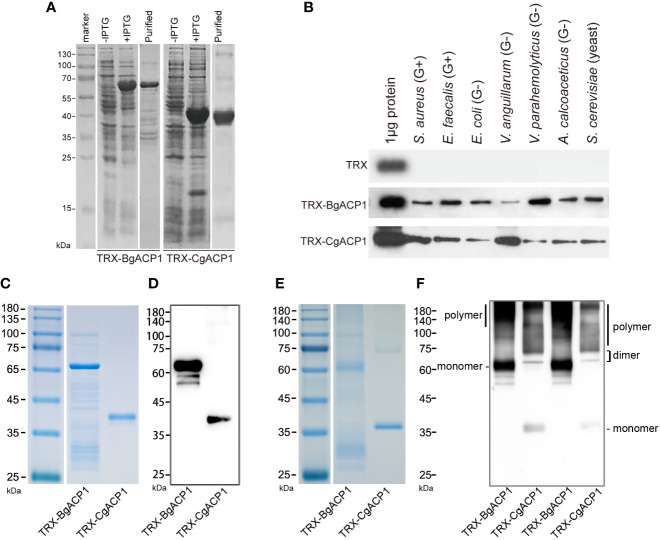
The bacterial binding capacity of recombinant CgACP1 and BgACP1. **(A)** SDS-PAGE analyses of samples collected during the purification of recombinant TRX-CgACP1 and TRX-BgACP1. **(B)** The binding of microbes by recombinant TRX-CgACP1 and TRX-BgACP1 protein. One representative experiment out of three is shown. **(C)** Coomassie Blue staining of the reduced SDS-PAGE of recombinant TRX-CgACP1 and TRX-BgACP1. **(D)** Western blotting of the reduced SDS-PAGE with anti-His Tag antibodies shows that multimerized recombinant TRX-CgACP1 and TRX-BgACP1 were broken down to a single band of monomers. **(E)** Coomassie Blue staining of the non-reduced SDS-PAGE of recombinant TRX-CgACP1 and TRX-BgACP1. **(F)** Western blotting of the non-reduced SDS-PAGE with anti-His Tag antibodies shows the bands of monomers, dimers and polymers of recombinant TRX-CgACP1 and TRX-BgACP1.

As secreted simple-structured proteins (especially CgACP1), both CgACP1 and BgACP1 might correspondingly have a simple, straightforward function such as recognizing microbes in the extracellular space. To investigate their molecular functions, we prepared and purified recombinant His-tagged TRX-CgACP1 and TRX-BgACP1 fusion proteins (**Figure 4A**). These recombinant proteins were then incubated with several microbes, including two gram-positive bacteria (*S. aureus* and *E. faecalis*), four gram-negative bacteria (*E. coli*, *V. anguillarum*, *V. parahemolyticus*, *A. calcoaceticus*) and a yeast (*S. cerevisiae*). After incubation, the microbial pellets were assessed by Western Blot using anti-His monoclonal antibodies (mAb), which shows that both TRX-CgACP1 and TRX-BgACP1 could bind with all selected microbes in different affinities (**Figure 4B**).

We then analyzed if the recombinant proteins could form multimers, as suggested by the conserved cysteines in the ApeC domain (**Figure 2C**). The intermolecular disulfide bonds could be broken down in a strong reducing condition. Indeed, we showed that in the reducing condition, both recombinant TRX-CgACP1 and TRX-BgACP1 were reduced to a single band of monomers, which are corresponding to their respectively molecular weights (**Figures 4C, D**). However, under a non-reducing condition in which disulfide bonds might be preserved, we observed the bands of monomers and dimers, as well as the high-weight smears which were supposed to be polymers (**Figures 4E, F**). This suggests that both recombinant TRX-CgACP1 and TRX-BgACP1 were capable of forming multimers through disulfide bonds.

**Figure 5:**
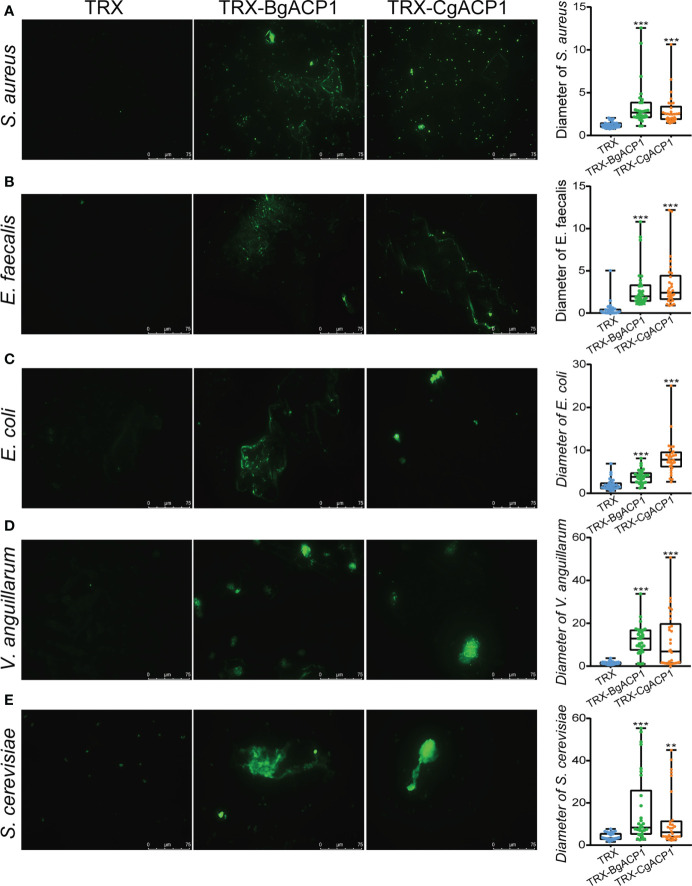
The bacterial agglutinating capacity of recombinant CgACP1 and BgACP1. **(A–E)** Agglutination of the different microbes by TRX-CgACP1 and TRX-BgACP1. The diameters of green puncta in microbial aggregation assays were measured by boxplot and shown on the right panels. Boxplot explanation: upper horizontal bar outside box, maximum value; lower horizontal bar outside box, minimum value; upper horizontal line of box, 75th percentile; lower horizontal line of box, 25th percentile; horizontal bar within box, median; **p < 0.01, ***p < 0.001 versus TRX control.

To further investigate whether the microbe-binding and multimerizing ability of BgACP1 and CgACP1 could allow them to aggregate the microbes, we incubated the recombinant proteins with FITC-labeled *S. aureus*, *E. faecalis*, *E. coli*, *V. anguillarum* and yeast *S. cerevisiae*, and then assessed the microbial aggregation by using fluorescence microscopy. The results show that the addition of TRX-CgACP1 and TRX-BgACP1 could cause observable aggregation of all the tested microbes. Quantification of the diameter of green microbial puncta confirmed the significance of the agglutination effects (**Figures 5A–E**). These results suggest that both CgACP1 and BgACP1 have broad-spectrum microbial binding and agglutinating capacities.

### Recombinant CgACP1 and BgACP1 showed no inhibition on bacterial growth

**Figure 6:**
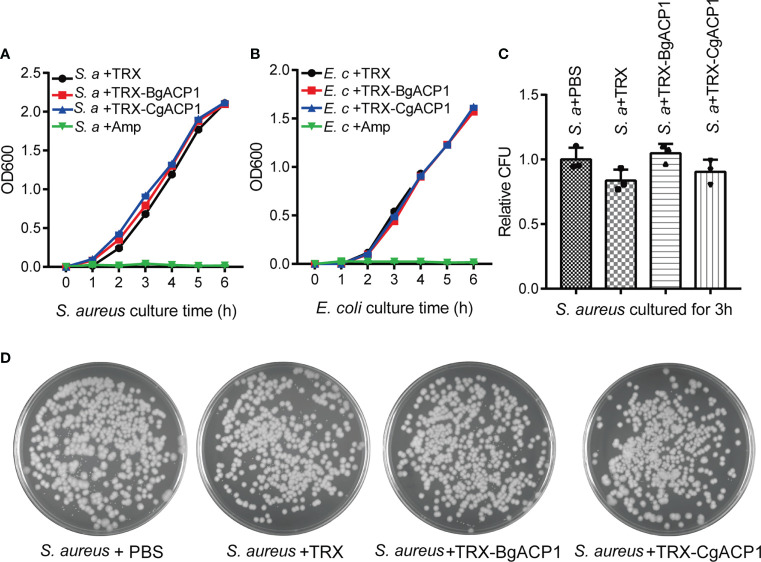
Recombinant CgACP1 and BgACP1 showed no inhibiting or killing activity to bacteria S. *aureus* and *E. coli.* Growth curves of *S. aureus* **(A)** and *E*. *coli* **(B)** in the presence of TRX-CgACP1 or TRX-GgACP1. 200 μg/ml of each ACP, Ampicillin (Amp) or TRX (as control) were incubated with the indicated bacteria and the OD_600_ was measured every 1 hour after starting the culture (mean ± SD, n = 3). **(C)** Mollusk ACPs did not affect the colony forming unit (CFU) of *S. aureus.* 200 μg/ml of each ACP or TRX (control) were incubated with the *S. aureus* for 3 hours and the numbers of bacteria were determined by colony counts. Relative CFU represents the ratio of CFU incubated with TRX/ACP and incubated with PBS. One of the three petri dishes used in **(C)** is shown in **(D)**.

Some secreted short-form bacteria-binding lectins, such as the human C-type lectin REG3 (40), are able to kill bacteria. To determine whether recombinant CgACP1 and BgACP1 could kill bacteria or interfere their growth, we monitored the growth curves of *S. aureus* and *E. coli* in the presence of the recombinant proteins. Antibiotic ampicillin was used as the positive control. The results showed that both TRX-CgACP1 and TRX-BgACP1 fusion protein had no inhibiting effects on the examined bacteria (**Figures 6A, B**). Moreover, the growth curves and the colony counting assays confirmed that both recombinant proteins could not kill bacteria *S. aureus* or *E. coli* (**Figures 6C, D**). As comparison, recombinant amphioxus ACP1/2/3/5 also showed no bactericidal activity (2, 6). We concluded that both CgACP1 and BgACP1 have microbial binding and aggregation activities, but might not have bactericidal and bacteriostatic ability.

### Recombinant CgACP1 and BgACP1 specifically recognized peptidoglycan

**Figure 7:**
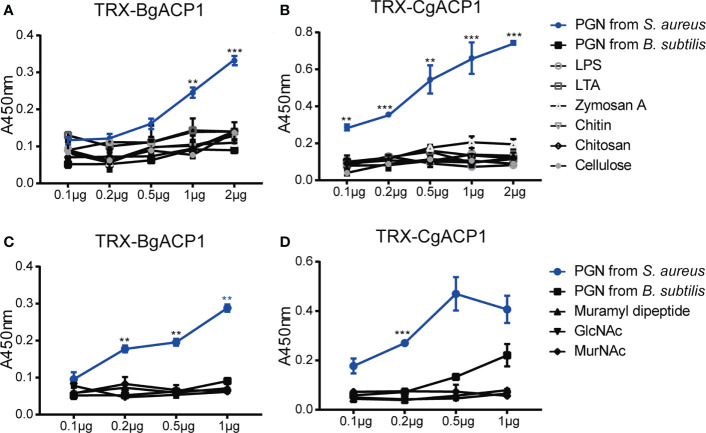
The carbohydrate-binding specificity and affinity of recombinant CgACP1 and BgACP1. **(A, B)** ELISA assays of the interaction between recombinant fusion TRX-CgACP1 and TRX-BgACP1 to different bacterial cell wall components. **(C, D)** ELISA assays of the interaction between recombinant fusion ACPs and several moieties of PGN. GlcNAc, N-acetylglucosamine; MurNAc, N-acetylmuramic acid. **p < 0.01, ***p < 0.001. Three biological replicates were conducted for each experiment, and three technical replicates were performed. One of the representative results was shown here.

Previous studies have shown that some amphioxus ApeC domains showed high binding affinity to certain microbial cell wall components (2, 6). To determine which microbial cell wall components could be recognized by CgACP1 and BgACP1, here we implemented ELISA assays as previously described (37). The results show that both recombinant CgACP1 and BgACP1 had much higher binding affinity with pepdicoglycan (PGN) than with lipopolysaccharide (LPS), lipoteichoicacid (LTA), Zymosan A, chitin, chitosan and cellulose (**Figures 7A, B**). PGN have two major structural types, namely, the Lys-type and the DAP-type. PGN from *S. aureus* is Lys-type while PGN from *B. subtilis* is DAP-type. Our ELISA assays further showed that both recombinant ACPs had high affinity to the Lys-type PGN from *S. aureus* but have trivial or even no affinity to DAP-type PGN from *B. subtilis* (**Figures 7A, B**).

PGN is a macromolecule composed of several heteropolysaccharides alternately linked by N-acetylglucosamine (GlcNAc), N-acetylmuramic acid (MurNAc) and cross-linked with peptides of different compositions. The PGN breakdown products could stimulate an inflammatory response. The products include GlcNAc and several dipeptide-containing motifs, such as muramyl dipeptide (MDP; MurNAc-L-Ala-D-isoGln) (41). MDP is the minimal bioactive moiety of PGN and the agonist of NOD2, which can activate the NOD2-mediated NF-κB and NLRP1 inflammasome (42–44) Here we showed that although recombinant CgACP1 and BgACP1 could bind with PGN, they showed no significant affinities to any of the PGN moieties, including MDP, GlcNAc and MurNAc (**Figures 7C, D**). We speculated that either the optimal affinity of CgACP1 and BgACP1 required a special conformation of PGN, or the recombinant CgACP1 and BgACP1 might not preserve certain binding ability in their native forms. Anyway, the binding specificities of CgACP1 and BgACP1 have both similarities and dissimilarities with those of amphioxus ACP1/2/3/5 (6).

## Discussion and conclusions

### Distribution, phylogenetics and diversity of the mollusk ACPs

ApeC is an invertebrate-specific protein domain and predominantly present in marine and freshwater invertebrates (5). Among those ApeC domain-containing phyla, *Mollusca* is the largest one. So far, a large number of ACPs (approximately one third of the known ACPs) are from mollusks, most of which are from gastropods and bivalves, the first and the second largest mollusk classes, respectively. Mollusk ACPs could be classified into at least nine phylogenetic groups. Mollusk ACPs have no orthologs in other phyla, and also appear to be more diversified and more actively duplicated in bivalves than in gastropods. Similar to the situations in amphioxus and other phyla, mollusk ApeC domains are present in various protein architectures, making them the versatile and promiscuous domains like the IG and C-type lectin domains (45, 46). In fact, each mollusk phylogenetic group has its own special protein architecture. Nevertheless, these sequence and structural diversities might reflect the diverse roles of ACPs in mollusks.

### Molecular functions of the mollusk ApeC domains

So far, the functions of ApeC were only investigated in several highly-expressed, secreted short-form ACPs from the amphioxus (2, 6). To facilitate cross-phylum comparison and to guarantee proper representation, here we selected two highly-expressed, secreted short-form mollusk ACPs for function investigation, with one from bivalves and the other from gastropods. We observed that both recombinant mollusk ACPs (CgACP1 and BgACP1) could bind and aggregate different microbes in different affinities. As comparison, amphioxus ACP1/2/3/5 also showed similar bacterial binding and aggregating capacities, though with different binding spectrum and affinity (2, 6). Moreover, both the two mollusk ACPs and the four amphioxus ACPs exhibited no inhibiting or killing effects on the tested microbes (2, 6).

In addition, we found that recombinant CgACP1 and BgACP1 could specifically bind to Lys-type PGN from *S. aureus* with high affinity, but had trivial or no affinity to DAP-type PGN from *B. subtilis* (**Figure 6**). As comparison, amphioxus ACP1 and ACP2 showed high affinity to Lys-type PGN and relatively low affinity to DAP-type PGN (2), whereas amphioxus ACP3 and ACP5 showed high affinity to Lys-type PGN but nearly no affinity to DAP-type PGN (6). Despite these difference, Lys-type PGN emerges to be the primary specific binding target of all examined ApeC domains so far.

Although Lys-type PGN had been identified as the main binding target of CgACP1 and BgACP1, both proteins could actually bind with G-negative bacteria and yeast which are supposed to have no Lys-type PGN. This inconsistence might be caused by several factors. First, these ACPs showed certain low level or background level of affinities to DAP-type PGN and other tested substances (**Figure 7**). Second, ApeC domains may have affinity to other unknown cell wall components. Third, there are differences in terms of the density of the target sites and their accessibility in the cell wall of different microbes, and different ACPs may have different ability to penetrate into the cell wall. Therefore, even if an ACP could recognize a cell wall component, they might bind and aggregate different bacteria with different strength. Fourth, the binding assays might not be conducted in the optimal conditions for CgACP1 and BgACP1. Nevertheless, the mechanisms underlying the binding specificity of ApeC domains require further research efforts.

### The role of short-form ACPs in the mucosal surface of the gill

Mollusk ACPs have been suggested to play important roles in immunity, development and stress resistance (10–12, 15–18). ACPs were even discovered in the pedal mucus of the limpets, suggesting a role in the glue-like adhesion (19). However, so far, there are no direct evidence to support any immune functions for the mollusk ApeC domains (10, 11, 15, 17). On the other hand, there are direct evidence from the amphioxus. Previous studies showed that the amphioxus ACP1 had the highest expression among all amphioxus ACPs and was mainly expressed in the gill, which is the filter feeding organ of amphioxus. ACP1 could be further up-regulated by hundreds of times in response to bacterial infection, and has been shown to act as an essential mucus lectin to defense the gill surface (2, 3, 47). In the normal physiological condition, if amphioxus ACP1 protein was neutralized, it would cause the disintegration of the gill due to bacterial invasion (2).

As comparison, here we showed that in pacific oyster, CgACP1 had the highest expression level of all oyster ACPs. CgACP1 was also predominantly expressed in the gill and labial palp in a very high level which is comparable to that of the beta-actin. This expression level could be greatly up-regulated quickly after bacterial infection. Remarkably, the gill and the labial palp are adjacent to each other and comprise the filter feeding organ for oysters, which is contacting the water environment, and continuously and non-selectively sieving the running water for small particles, including algae, bacteria, other food particles and even pathogens. In addition, CgACP1 and amphioxus ACP1 are both typical short-form ACPs and have similar binding properties. In the sense, CgACP1 could play a similar role in the gill and in the filter feeding process as the amphioxus ACP1.

### Basic functional properties of the ApeC domains

In this study, we provided a survey of the mollusk ACPs in terms of their species distribution, composition, phylogenetics, structures and expression patterns. Based on this survey, we selected two highly-expressed, secreted short-form ACPs as the representative mollusk ACPs for the further functional survey. By comparing the findings in this study and in the previous studies of amphioxus ACP1/2/3/5, and by referring to the indirect evidence from other related studies, we could identify several functional traits of the ApeC domains. These traits have been shown to be conserved across different phyla, including the high affinity to Lys-type PGN, the bacterial binding and agglutinating capacity, and the role as mucus lectin-type pattern-recognition proteins in the mucosal surface. In conclusion, this may not only extend our understanding of the mollusk’s immune diversity, but guide our future researches of the ACP functions in mollusks as well as in other animal clades.

## Data availability statement

The original contributions presented in the study are included in the article/**Supplementary Material**. Further inquiries can be directed to the corresponding authors.

## Author contributions

SH, JL and AX designed the study. JL performed the experiments. JL, SL, YZ, FM, HF and QH analyzed the data. HZ, JO, WY, FM and MD also conducted some experiments and provided reagents, materials and some data explanation. JL, SH and AX drafted the manuscript. All authors discussed and agreed on the results and approved the manuscript.

## Funding

This work was supported by the project from Southern Marine Science and Engineering Guangdong Laboratory (Zhuhai) (SML2021SP304), National Key R&D Program of China (2018YFD0900503), National Science Foundation of China (NNSF) Projects (31872595, 32073002 and 31722052), projects from Guangdong and Guangzhou (2021A1515012380 and 2020B1212060031), and Fundamental Research Funds for the Central Universities (Sun Yat-sen University; 22lglj09).

## Conflict of interest

The authors declare that the research was conducted in the absence of any commercial or financial relationships that could be construed as a potential conflict of interest.

## Publisher’s note

All claims expressed in this article are solely those of the authors and do not necessarily represent those of their affiliated organizations, or those of the publisher, the editors and the reviewers. Any product that may be evaluated in this article, or claim that may be made by its manufacturer, is not guaranteed or endorsed by the publisher.
