# Supplementary material for: Two novel mollusk short-form ApeC-containing proteins act as pattern recognition proteins for peptidoglycan
Source: Front Immunol. 2022 Oct 7;13:971883. doi: 10.3389/fimmu.2022.971883 (PMC9585378; doi:10.3389/fimmu.2022.971883)
Supplement: Supplementary file 1 [file DataSheet_1.pdf]

Supplementary Table 1. Accession numbers of the ACPs used for phylogenetic analysis. 2

Supplementary Table 2. RNA-seq counts of representative invertebrate short-form ACPs from NCBI. 6

Supplementary Table 3. RNA-seq data of CgACP1 in different tissues from BioProject PRJNA146329 of NCBI. 7

Supplementary Table 4. RNA-seq data of CgACP1 after Vibrio challenge from BioProject PRJNA194079 of NCBI. 8

Supplementary Figure 1. The Maximum Likelihood tree of all ACPs from mollusks based on the ApeC domain. 9

Supplementary Figure 2. Nucleotide and deduced amino acid sequences of CgACP1 and BgACP1. 10

Supplementary Table 1. Accession numbers of the ACPs used for phylogenetic analysis.

Phylum	Class	Species	Accession number
Mollusca	Gastropoda	*Aplysia californica*	XP_012940199.1
Mollusca	Gastropoda	*Aplysia californica*	XP_012936733.1
Mollusca	Gastropoda	*Aplysia californica*	XP_005092781.2
Mollusca	Gastropoda	*Aplysia californica*	XP_005088874.2
Mollusca	Gastropoda	*Aplysia californica*	XP_005108722.1
Mollusca	Gastropoda	*Aplysia californica*	XP_005103935.1
Mollusca	Gastropoda	*Aplysia californica*	XP_005096035.1
Mollusca	Gastropoda	*Aplysia californica*	XP_035826903.1
Mollusca	Gastropoda	*Biomphalaria glabrata*	XP_013095350.1
Mollusca	Gastropoda	*Biomphalaria glabrata*	XP_013078768.1
Mollusca	Gastropoda	*Biomphalaria glabrata*	XP_013070847.1
Mollusca	Gastropoda	*Biomphalaria glabrata*	XP_013095349.1
Mollusca	Gastropoda	*Biomphalaria glabrata*	XP_013075037.1
Mollusca	Gastropoda	*Biomphalaria glabrata*	XP_013075036.1
Mollusca	Gastropoda	*Lottia gigantea*	XP_009062790.1
Mollusca	Gastropoda	*Lottia gigantea*	XP_009056306.1
Mollusca	Gastropoda	*Lottia gigantea*	XP_009050301.1
Mollusca	Gastropoda	*Lottia gigantea*	XP_009047393.1
Mollusca	Gastropoda	*Lottia gigantea*	XP_009045922.1
Mollusca	Gastropoda	*Plakobranchus ocellatus*	GFO21125.1
Mollusca	Gastropoda	*Plakobranchus ocellatus*	GFN94188.1
Mollusca	Gastropoda	*Plakobranchus ocellatus*	GFN94187.1
Mollusca	Gastropoda	*Batillaria attramentaria*	KAG5708631.1
Mollusca	Gastropoda	*Elysia chlorotica*	RUS72794.1
Mollusca	Gastropoda	*Pomacea canaliculata*	XP_025100017.1
Mollusca	Gastropoda	*Gigantopelta aegis*	XP_041366864.1
Mollusca	Gastropoda	*Gigantopelta aegis*	2034332671
Mollusca	Gastropoda	*Gigantopelta aegis*	XP_041366865.1
Mollusca	Gastropoda	*Patella vulgata*	QKY77478.1
Mollusca	Bivalvia	*Crassostrea gigas*	XP_034333817.1
Mollusca	Bivalvia	*Crassostrea gigas*	XP_034306860.1
Mollusca	Bivalvia	*Crassostrea gigas*	XP_034306861.1
Mollusca	Bivalvia	*Crassostrea gigas*	XP_034306864.1
Mollusca	Bivalvia	*Crassostrea gigas*	XP_034306862.1
Mollusca	Bivalvia	*Crassostrea gigas*	XP_034304086.1
Mollusca	Bivalvia	*Crassostrea gigas*	XP_034305119.1
Mollusca	Bivalvia	*Crassostrea gigas*	XP_034305128.1
Mollusca	Bivalvia	*Crassostrea gigas*	XP_034305131.1
Mollusca	Bivalvia	*Crassostrea gigas*	XP_034305121.1
Mollusca	Bivalvia	*Crassostrea gigas*	XP_034304087.1
Mollusca	Bivalvia	*Crassostrea gigas*	XP_034306863.1

Supplementary Table 1 (*continued*)

Phylum	Class	Species	Accession number
Mollusca	Bivalvia	*Crassostrea gigas*	XP_034305129.1
Mollusca	Bivalvia	*Crassostrea gigas*	XP_034339231.1
Mollusca	Bivalvia	*Crassostrea gigas*	XP_034311325.1
Mollusca	Bivalvia	*Crassostrea gigas*	XP_034305976.1
Mollusca	Bivalvia	*Crassostrea gigas*	XP_034305975.1
Mollusca	Bivalvia	*Crassostrea gigas*	XP_034305977.1
Mollusca	Bivalvia	*Crassostrea gigas*	XP_011435747.2
Mollusca	Bivalvia	*Crassostrea gigas*	XP_011431415.2
Mollusca	Bivalvia	*Crassostrea gigas*	XP_011455971.2
Mollusca	Bivalvia	*Mizuhopecten yessoensis*	XP_021372494.1
Mollusca	Bivalvia	*Mizuhopecten yessoensis*	XP_021343358.1
Mollusca	Bivalvia	*Mizuhopecten yessoensis*	XP_021343350.1
Mollusca	Bivalvia	*Mizuhopecten yessoensis*	XP_021365717.1
Mollusca	Bivalvia	*Mizuhopecten yessoensis*	XP_021353768.1
Mollusca	Bivalvia	*Mizuhopecten yessoensis*	XP_021348426.1
Mollusca	Bivalvia	*Mizuhopecten yessoensis*	OWF42800.1
Mollusca	Bivalvia	*Mizuhopecten yessoensis*	OWF35041.1
Mollusca	Bivalvia	*Mizuhopecten yessoensis*	OWF35016.1
Mollusca	Bivalvia	*Mytilus galloprovincialis*	OPL33621.1
Mollusca	Bivalvia	*Mytilus galloprovincialis*	OPL32926.1
Mollusca	Bivalvia	*Mytilus galloprovincialis*	OPL21662.1
Mollusca	Bivalvia	*Mytilus galloprovincialis*	AJQ21523.1
Mollusca	Bivalvia	*Mytilus galloprovincialis*	AJQ21519.1
Mollusca	Bivalvia	*Mytilus galloprovincialis*	AJQ21517.1
Mollusca	Bivalvia	*Mytilus galloprovincialis*	AKQ70860.1
Mollusca	Bivalvia	*Mytilus galloprovincialis*	AEK10750.1
Mollusca	Bivalvia	*Mytilus galloprovincialis*	AEK10749.1
Mollusca	Bivalvia	*Mytilus galloprovincialis*	VDI26908.1
Mollusca	Bivalvia	Mytilus galloprovincialis	VDI26909.1
Mollusca	Bivalvia	*Mytilus galloprovincialis*	VDI17271.1
Mollusca	Bivalvia	*Mytilus galloprovincialis*	VDI37935.1
Mollusca	Bivalvia	*Mytilus galloprovincialis*	VDI32706.1
Mollusca	Bivalvia	*Mytilus galloprovincialis*	VDI18533.1
Mollusca	Bivalvia	*Mytilus galloprovincialis*	VDI37936.1
Mollusca	Bivalvia	Mytilus galloprovincialis	VDH95057.1
Mollusca	Bivalvia	*Mytilus galloprovincialis*	VDH95058.1
Mollusca	Bivalvia	*Mytilus galloprovincialis*	VDI16311.1
Mollusca	Bivalvia	*Mytilus galloprovincialis*	VDI16310.1
Mollusca	Bivalvia	*Mytilus galloprovincialis*	VDI49239.1
Mollusca	Bivalvia	*Mytilus galloprovincialis*	VDI49238.1
Mollusca	Bivalvia	*Mytilus galloprovincialis*	VDI32707.1
Mollusca	Bivalvia	*Mytilus galloprovincialis*	VDH98273.1

Supplementary Table 1 (*continued*)

Phylum	Class	Species	Accession number
Mollusca	Bivalvia	*Mytilus galloprovincialis*	OPL20997.1
Mollusca	Bivalvia	*Mytilus coruscus*	CAC5419765.1
Mollusca	Bivalvia	*Mytilus coruscus*	CAC5418400.1
Mollusca	Bivalvia	*Mytilus coruscus*	CAC5400412.1
Mollusca	Bivalvia	*Mytilus coruscus*	CAC5376172.1
Mollusca	Bivalvia	*Mytilus coruscus*	CAG2254798.1
Mollusca	Bivalvia	*Mytilus coruscus*	CAC5392656.1
Mollusca	Bivalvia	*Mytilus coruscus*	CAC5419767.1
Mollusca	Bivalvia	*Mytilus coruscus*	CAC5419766.1
Mollusca	Bivalvia	*Mytilus edulis*	CAG2226732.1
Mollusca	Bivalvia	*Mytilus edulis*	CAG2184481.1
Mollusca	Bivalvia	*Mytilus edulis*	CAG2214854.1
Mollusca	Bivalvia	*Mytilus edulis*	CAG2254797.1
Mollusca	Bivalvia	*Mytilus edulis*	AG2248606.1
Mollusca	Bivalvia	*Mytilus edulis*	CAG2197871.1
Mollusca	Bivalvia	*Mytilus edulis*	CAG2230279.1
Mollusca	Bivalvia	*Mytilus edulis*	CAG2239526.1
Mollusca	Bivalvia	*Mytilus edulis*	CAG2214855.1
Mollusca	Bivalvia	*Mytilus edulis*	CAG2214847.1
Mollusca	Bivalvia	*Mytilus edulis*	CAG2199568.1
Mollusca	Bivalvia	*Mytilus edulis*	CAG2239191.1
Mollusca	Bivalvia	*Crassostrea virginica*	XP_022290608.1
Mollusca	Bivalvia	*Crassostrea virginica*	XP_022289832.1
Mollusca	Bivalvia	*Crassostrea virginica*	XP_022296255.1
Mollusca	Bivalvia	*Crassostrea virginica*	XP_022300142.1
Mollusca	Bivalvia	*Crassostrea virginica*	XP_022300279.1
Mollusca	Bivalvia	*Crassostrea virginica*	XP_022311533.1
Mollusca	Bivalvia	*Crassostrea virginica*	P_022296259.1
Mollusca	Bivalvia	*Crassostrea virginica*	XP_022296260.1
Mollusca	Bivalvia	*Crassostrea virginica*	XP_022302103.1
Mollusca	Bivalvia	*Crassostrea virginica*	XP_022301903.1
Mollusca	Bivalvia	*Crassostrea virginica*	XP_022296258.1
Mollusca	Bivalvia	*Crassostrea virginica*	XP_022300281.1
Mollusca	Bivalvia	*Crassostrea virginica*	XP_022298832.1
Mollusca	Bivalvia	*Crassostrea virginica*	XP_022300141.1
Mollusca	Bivalvia	*Crassostrea virginica*	XP_022300140.1
Mollusca	Bivalvia	*Crassostrea virginica*	XP_022296256.1
Mollusca	Bivalvia	*Crassostrea virginica*	XP_022302246.1
Mollusca	Bivalvia	*Crassostrea virginica*	XP_022322789.1
Mollusca	Bivalvia	*Dreissena polymorpha*	KAH3701576.1
Mollusca	Bivalvia	*Dreissena polymorpha*	KAH3701576.1
Mollusca	Bivalvia	*Pecten maximus*	XP_033758459.1

Supplementary Table 1 (*continued*)

**Phylum**	**Class**	**Species**	**Accession number**
Mollusca	Bivalvia	*Pecten maximus*	XP_033760733.1
Mollusca	Bivalvia	*Pecten maximus*	XP_033745945.1
Mollusca	Bivalvia	*Pecten maximus*	XP_033750083.1
Mollusca	Bivalvia	*Pecten maximus*	XP_033755203.1
Mollusca	Cephalopoda	*Octopus sinensis*	XP_036368646.1

Supplementary Table 2. RNA-seq counts of representative invertebrate short-form ACPs from NCBI.

Phylum	Species	Accession number	RNA-seq counts (reads counts)
Echinodermata	*Strongylocentrotus purpuratus*	XP_794322	73906
Echinodermata	*Strongylocentrotus purpuratus*	XP_001200514	41476
Echinodermata	*Strongylocentrotus purpuratus*	XP_011683558	17318
Echinodermata	*Strongylocentrotus purpuratus*	XP_001200514	41476
Hemichordata	*Saccoglossus kowalevskii*	XP_002735920.1	10
Mollusca	*Aplysia_californica*	XP_012940199.1	333723
Mollusca	*Aplysia_californica*	XP_012946907.1	125
Mollusca	*Aplysia_californica*	XP_005108722.1	854
Mollusca	*Aplysia_californica*	XP_005103935.1	7297
Mollusca	*Aplysia_californica*	XP_005096035.1	133873
Mollusca	*Biomphalaria glabrata*	XP_013095350	2369
Mollusca	*Biomphalaria glabrata*	XP_013078768	29328
Mollusca	*Biomphalaria glabrata*	XP_013070847	9281
Mollusca	*Crossostrea gigas*	XP_011449958	39645
Mollusca	*Crossostrea gigas*	XP_011457183	14550
Mollusca	*Crossostrea gigas*	XP_011431415	18730
Mollusca	*Crossostrea gigas*	XP_011455453	1009
Mollusca	*Crossostrea gigas*	XP_011455971	95552
Mollusca	*Crossostrea gigas*	XP_034306861	554224
Mollusca	*Crossostrea gigas*	XP_011421556	543914
Cephalochordata	*Branchiostoma belcheri*	XP_019618988	15886
Cephalochordata	*Branchiostoma belcheri*	XP_019620972	15886
Cephalochordata	*Branchiostoma belcheri*	XP_019626105	211
Cephalochordata	*Branchiostoma belcheri*	XP_019621313	9246
Cnidaria	*Acropora digitifera*	XP_015764543	724
Cnidaria	*Acropora digitifera*	XP_015762623	2241
Cnidaria	*Acropora digitifera*	XP_015762523	246290
Cnidaria	*Acropora digitifera*	XP_015758720	3902
Cnidaria	*Acropora digitifera*	XP_015754320	969
Cnidaria	*Nematostella vectensis*	XP_001632794	43633
Cnidaria	*Nematostella vectensis*	XP_001630625	1097347
Cnidaria	*Nematostella vectensis*	XP_001628165	36468
Cnidaria	*Orbicella faveolata*	XP_020601927	612
Cnidaria	*Orbicella faveolata*	XP_020627719	263
Cnidaria	*Orbicella faveolata*	XP_020630203	993

Supplementary Table 3. RNA-seq data of CgACP1 in different tissues from BioProject PRJNA146329 of NCBI.

			FPKM values		
**tissue**	**BioSample**	**Run**	**ACP1**	**Actin**	**Gapdh**
outer edge of mantle	SAMN00714285	SRR334212	284.8	4409.6	212.8
digestive gland	SAMN00714286	SRR334213	73.0	642.3	105.7
female gonad	SAMN00714287	SRR334214	67.3	445.7	201.5
gill	SAMN00714288	SRR334215	1308.3	1120.9	174.2
inner part of mantle	SAMN00714289	SRR334216	127.3	5943.5	186.7
adductor muscle	SAMN00714290	SRR334217	29.5	6283.3	382.6
hemolymph	SAMN00714291	SRR334218	33.8	657.8	311.3
labial palp	SAMN00714292	SRR334219	355.7	740.7	111.4
male gonad	SAMN00714293	SRR334220	244.2	1091.1	191.8

FPKM = Fragments Per Kilobase of exon model per Million mapped fragments

Supplementary Table 4. RNA-seq data of CgACP1 after Vibrio challenge from BioProject PRJNA194079 of NCBI.

				FPKM values		
**tissue**	**Sample name**	**BioSample**	**Run**	**ACP1**	**Actin**	**Gapdh**
gill	0h after *Vibrio* challenge	SAMN01986138	SRR796582	1161.0	774.5	147.0
gill	6h after *Vibrio* challenge	SAMN01986139	SRR796584	1536.5	860.3	92.0
gill	12h after *Vibrio* challenge	SAMN01986140	SRR796583	1032.3	798.1	121.2
gill	24h after *Vibrio* challenge	SAMN01986141	SRR796585	1019.6	624.2	133.9
gill	48h after *Vibrio* challenge	SAMN01986142	SRR796586	786.6	746.8	132.6
gill	Control, no injection	SAMN01986149	SRR796589	318.9	726.8	140.3

FPKM = Fragments Per Kilobase of exon model per Million mapped fragments

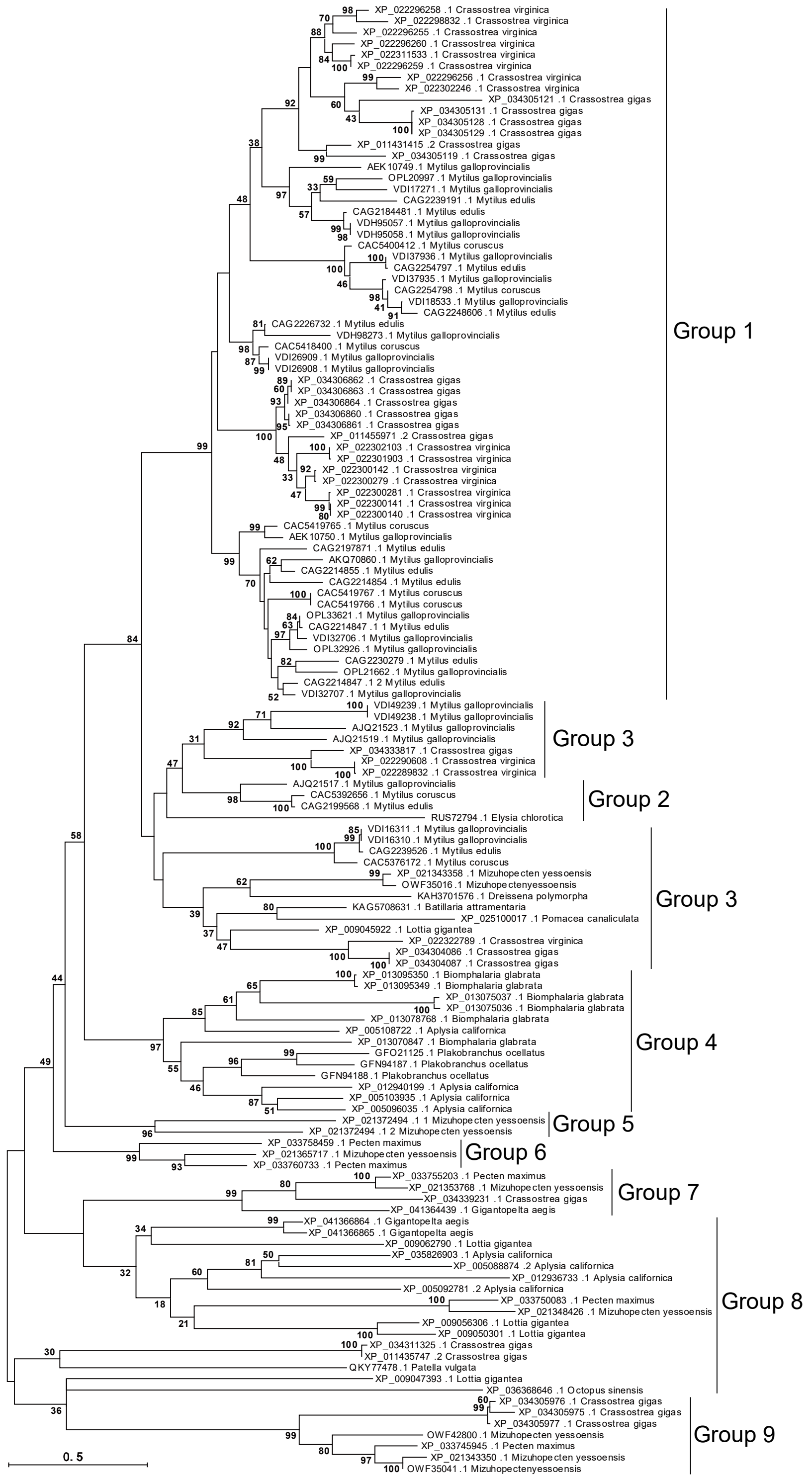

Supplementary Figure 1. The Maximum Likelihood tree of all ACPs from mollusks based on the ApeC domain.

Evolutionary analyses were conducted in MEGA-X with WAG model, Gamma distribution of rates across sites model and 1000 bootstrap tests. The percentage of trees in which the associated taxa clustered together is shown next to the branches. The tree is drawn to scale, with branch lengths measured in the number of substitutions per site.

A

```
    TTTAACGGTCTAAAGGTCCTGGACACAATGGAGGGACTAGTCTTTATCCTAGCCGTGCTAAGTCTCTGGGTTGCA
1                                M  E  G  L  V  F  I  L  A  V  L  S  L  W  V  A
    TCCTCTTCTGCAGTCGGATGGCCTTCTGGAACGTACTCAATGATAACCCCTAAAGTTGGGTGCCCTTCCGGATGG
26   S  S  S  A  V  G  W  P  S  G  T  Y  S  M  I  T  P  K  V  G  C  P  S  G  W
    AAATTGGGCTGGCGGTACCAGGATAATGAGGACACTGGAAATATTAACTCAATGACCAGTAATCATCACTTCAAT
51   K  L  G  W  R  Y  Q  D  N  E  D  T  G  N  I  N  S  M  T  S  N  H  H  F  N
    GGATTCTTTTTCGATGACACAATTCATTATTACTGCTCCAAAACCTCGTACTCTGGATCTGGATCATGGCCTCGA
76   G  F  F  F  D  D  T  I  H  Y  Y  C  S  K  T  S  Y  S  G  S  G  S  W  P  R
    GGAAACTACTGCATCATGAGATATGGCTCTTCCTGTCCTTCAGGTTTCAGTTCGGGCAGTATTTACTGGGATGAC
101  G  N  Y  C  I  M  R  Y  G  S  S  C  P  S  G  F  S  S  G  S  I  Y  W  D  D
    GAGGATTCCTACAATATGAATGGGAAGGGGGGATATGTACCCTCCGGGACTTATGGCTCTAATACACGAATCAAC
126  E  D  S  Y  N  M  N  G  K  G  G  Y  V  P  S  G  T  Y  G  S  N  T  R  I  N
    TACTGTTGCAGGAGTGACGGAAGCGCCTCCTCTTACATTTCACTCCCTACCACGGATCCGTTCTATCTGATGCGT
151  Y  C  C  R  S  D  G  S  A  S  S  Y  I  S  L  P  T  T  D  P  F  Y  L  M  R
    TACACATCCACAATATGCCAGAGGGTCAGTGGCATGACTGTTAGAGAGGAGATCATAACAACGGACGATGAGGAC
176  Y  T  S  T  I  C  Q  R  V  S  G  M  T  V  R  E  E  I  I  T  T  D  D  E  D
    ACATCTAACAATAACTCAGTGTCCGGAAGTCATCCCAAGGTTACCGGAACCAGAAACCATCGTCTCTACTACTGC
201  T  S  N  N  N  S  V  S  G  S  H  P  K  V  T  G  T  R  N  H  R  L  Y  Y  C
    TACTACAGTTAGGAAGGCAAATGTGTAATCTCT
226  Y  Y  S  *
```

B

```
    ATGTCGTGCAAGTTTGCATTCTTGCTGTCCTCTTTTTGTTGTTTTGCCGCCATTGAGGCCTCATTCTTGGTGACC
1    M  S  C  K  F  A  F  L  L  S  S  F  C  C  F  A  A  I  E  A  S  F  L  V  T
    ATCAACCCTACAAGAGTAGAAACAGGCATTACCCATAATGTGACAGTCACTTGCAGCTACTGGGGAGAGAGCTCT
26   I  N  P  T  R  V  E  T  G  I  T  H  N  V  T  V  T  C  S  Y  W  G  E  S  S
    TTAAGTTCTCTTGAGAAGATCACAATGATCCGCATCTTGCACCAGAAAACGACGGAGCCTTTCTCTGTGGTGGCG
51   L  S  S  L  E  K  I  T  M  I  R  I  L  H  Q  K  T  T  E  P  F  S  V  V  A
    GAGATACGAGACAACGAGAACAACGTCCACACCTCCGGCCTGAATTCCGGCGTCGTTGTGTCCGGTGCAATCAAG
76   E  I  R  D  N  E  N  N  V  H  T  S  G  L  N  S  G  V  V  V  S  G  A  I  K
    ACCCTTGATGATTCGTTCGTTGAGATTCTATGGCCCGTGGCCACACCTGATCTCTATGGTCACTATCGGTGTGAC
101  T  L  D  D  S  F  V  E  I  L  W  P  V  A  T  P  D  L  Y  G  H  Y  R  C  D
    GTAATCGGCTTCTCGATCACCCAAGACATCATCACGGAGAAGTCGCCCATCGTGTCCATCCTGGAGACGAACGTC
126  V  I  G  F  S  I  T  Q  D  I  I  T  E  K  S  P  I  V  S  I  L  E  T  N  V
    ACAGCCAACGACGTGCTGGAACTGTTGATCAGGGAGAAGGAAGAAATGAAGAGCTACTGCGATGCCAGGGTGGCG
151  T  A  N  D  V  L  E  L  L  I  R  E  K  E  E  M  K  S  Y  C  D  A  R  V  A
    GCTTCCGAAAGCAACTTCCAGACGCTGCTGAACAACCTGAGGAGGGAAGAGGCCAGCAACAATGACATCCTGGAG
176  A  S  E  S  N  F  Q  T  L  L  N  N  L  R  R  E  E  A  S  N  N  D  I  L  E
    AACACGTTGAGGGGAGAGCTGTCTGCGCTGAGAGAGGATGTCAACAGGCTGATGGAGACCGGAGTCCTCCAGTAC
201  N  T  L  R  G  E  L  S  A  L  R  E  D  V  N  R  L  M  E  T  G  V  L  Q  Y
    TGGCCTGAAGGCACGTATGCTCTTCTATCGCCTCAAGCAGGATGCCCGAACAACGTTGGAGCCATCTGGACAACA
226  W  P  E  G  T  Y  A  L  L  S  P  Q  A  G  C  P  N  N  V  G  A  I  W  T  T
    GGCTTAGTCAAGATTCACACTGAGTCGACTGACCGGAATTACGACCAGGTTTCAACAAATTCCCATCTTTTGGCT
251  G  L  V  K  I  H  T  E  S  T  D  R  N  Y  D  Q  V  S  T  N  S  H  L  L  A
    CCTATCCTTGAAAGGGCCGGAACGAACAACTTCATGTATCAACACTTCTGTGTTTCAACCACTCTCTCCCCAGGA
276  P  I  L  E  R  A  G  T  N  N  F  M  Y  Q  H  F  C  V  S  T  T  L  S  P  G
    GCGGCTTGGCCGAAAGGATCTTACTGTATCAACCGCAAGGGAGATAAATGCCCGACTGGATTCGAAACCGGATCA
301  A  A  W  P  K  G  S  Y  C  I  N  R  K  G  D  K  C  P  T  G  F  E  T  G  S
    ATCCAGTGGAACGAAGAAAGAACCGGCTCCGCGGGTGCCACCACAGGAGCTATTCCTGACGGCACCTATACAGCA
326  I  Q  W  N  E  E  R  T  G  S  A  G  A  T  T  G  A  I  P  D  G  T  Y  T  A
    GCTATTGCCAAAATATTTTACTGCTGCCGCAACGACAGCGACCCTTTCCACCCCGTCTACCTCCCGAAAGCCCGC
351  A  I  A  K  I  F  Y  C  C  R  N  D  S  D  P  F  H  P  V  Y  L  P  K  A  R
    CCCTTCTACTTATACAGATTCAAGGGCACATGCCAGGATGTGGTGGGCATGAAGGTCACTCCCGAGCTCATGGTC
376  P  F  Y  L  Y  R  F  K  G  T  C  Q  D  V  V  G  M  K  V  T  P  E  L  M  V
    TTCGACACGGACACCACTAATGCTGATGCCTATGACAATGACTGGCATCCAGACGGTCAGATCAACGATGTCCAC
401  F  D  T  D  T  T  N  A  D  A  Y  D  N  D  W  H  P  D  G  Q  I  N  D  V  H
    TTACTGCTGTGCTATTACGAAGAACGAGTTTAG
426  L  L  L  C  Y  Y  E  E  R  V  *
```

Supplementary Figure 2. Nucleotide and deduced amino acid sequences of CgACP1 and BgACP1.

Nucleotide and deduced amino acid sequences of CgACP1 **(A)** and BgACP1 (**B**). The start codon (ATG) and the stop codon (TAA) are in bold. The signal peptide predicted by SMART are underlined, highlighted in italics and the ApeC domain predicted by SMART are highlighted in blue background. The eight conserved Cysteine residues are in bold and red, and the three relatively conserved DXED motifs are bold.
